# Mechanisms Involved in Microglial-Interceded Alzheimer’s Disease and Nanocarrier-Based Treatment Approaches

**DOI:** 10.3390/jpm11111116

**Published:** 2021-10-29

**Authors:** Shadab Md, Nabil A. Alhakamy, Mohamed A. Alfaleh, Obaid Afzal, Abdulmalik S. A. Altamimi, Ashif Iqubal, Rasheed A. Shaik

**Affiliations:** 1 Department of Pharmaceutics, Faculty of Pharmacy, King Abdulaziz University, Jeddah 21589, Saudi Arabia; nalhakamy@kau.edu.sa (N.A.A.); maalfaleh@kau.edu.sa (M.A.A.); 2 Center of Excellence for Drug Research & Pharmaceutical Industries, King Abdulaziz University, Jeddah 21589, Saudi Arabia; 3 Vaccines and Immunotherapy Unit, King Fahd Medical Research Center, King Abdulaziz University, Jeddah 21589, Saudi Arabia; 4 Department of Pharmaceutical Chemistry, College of Pharmacy, Prince Sattam Bin Abdulaziz University, Al-Kharj 11942, Saudi Arabia; obaid263@gmail.com (O.A.); as.altamimi@psau.edu.sa (A.S.A.A.); 5 Department of Pharmacology, School of Pharmaceutical Education and Research, Jamia Hamdard, New Delhi 110062, India; asifiqubal2013@gmail.com; 6 Department of Pharmacology & Toxicology, Faculty of Pharmacy, King Abdulaziz University, Jeddah 21589, Saudi Arabia; rashaikh1@kau.edu.sa

**Keywords:** glial cells, neuroinflammation, dementia, signaling pathways, immunopathology, nanocarriers

## Abstract

Alzheimer’s disease (AD) is a common neurodegenerative disorder accountable for dementia and cognitive dysfunction. The etiology of AD is complex and multifactorial in origin. The formation and deposition of amyloid-beta (Aβ), hyperphosphorylated tau protein, neuroinflammation, persistent oxidative stress, and alteration in signaling pathways have been extensively explored among the various etiological hallmarks. However, more recently, the immunogenic regulation of AD has been identified, and macroglial activation is considered a limiting factor in its etiological cascade. Macroglial activation causes neuroinflammation via modulation of the NLRP3/NF-kB/p38 MAPKs pathway and is also involved in tau pathology via modulation of the GSK-3β/p38 MAPK pathways. Additionally, microglial activation contributes to the discrete release of neurotransmitters and an altered neuronal synaptic plasticity. Therefore, activated microglial cells appear to be an emerging target for managing and treating AD. This review article discussed the pathology of microglial activation in AD and the role of various nanocarrier-based anti-Alzeihmenr’s therapeutic approaches that can either reverse or inhibit this activation. Thus, as a targeted drug delivery system, nanocarrier approaches could emerge as a novel means to overcome existing AD therapy limitations.

## 1. Introduction

Alzheimer’s disease (AD) is a common neurodegenerative disorder. AD patients experience a poor quality of life and often remain unresponsive to most therapeutic regimens. Among the various causes of dementia, AD alone is responsible for more than 70% of cases [1]. In general, AD is diagnosed among older people, but recent evidence has shown the pathogenesis of AD among adult patients. It is reported that the total registered AD cases in the 1990s were approximately 20.2 million. In contrast, by the end of 2020, the number was more than double (about 50 million), and it is expected that by the end of 2050, cases may reach 125 million [2,3]. Poor cognitive function, dementia, behavioral abnormalities, attention deficit, social withdrawal syndrome, and verbal dysfunction are common clinical manifestations of AD [4]. These symptoms worsen the patients’ quality of life and impose enormous social and economic burdens on society and the nation [4]. Based on advancements in diagnostic and analytical techniques, several risk factors have been identified as causative agents of AD, such as increased exposure to environmental toxicants, genetic factors, mutations, trauma, and metabolic diseases, including diabetes mellitus and obesity [5].

The most common targets for the treatment and management of AD are based on the molecular pathogenesis of AD, including the accumulation of Aβ, a dysfunctional cholinergic system, the presence of neurofibrillary tangles (NFT), increased oxidative stress, persistent neuroinflammation, and microglia activation [6]. However, among the factors mentioned above, the presence of Aβ, NFT, and neuroinflammation involving microglial activation are extensively used to design targeted-based therapies [7]. Furthermore, it is important to highlight that neuroinflammation is considered the checkpoint for NFT and Aβ production. Hence, it is proposed that a targeted therapy for taking care of the neuroinflammatory aspect of AD could be an alternative approach [5]. Nevertheless, drugs approved by the food and drug administration (FDA), such as rivastigmine, galantamine, donepezil, memantine, and tacrine, have shown mild anti-Alzheimer’s effects [8].

Studies have shown that almost half of patients showed beneficial therapeutic outcomes when treated with these approved drugs. Unfortunately, there are severe peripheral and nervous side effects with their administration [9]. Furthermore, these drugs only reduce cognitive dysfunction and progression but are devoid of any impact on the cure of AD. Therefore, the current focus has shifted towards developing a targeted-based therapy (TBT) that will act on the pathological checkpoint of the disease and ensure maximum bioavailability at the site of action [7,9]. As discussed above, neuroinflammation is one of the major checkpoints in AD’s pathogenesis, and microglial activation is a leading cause of neuroinflammation in AD [5]. Hence, in this manuscript, we discuss the etiology of microglial activation in AD, highlight the clinical evidence of microglial activation, discuss the various targeted-based therapies for microglial activation, and project the possible therapeutic involvement of various nanocarrier-based approaches against microglial activation. Nanocarriers and nano-formulation offer several advantages compared to the conventional therapeutic approaches, such as bypassing hepatic metabolism, reduced dose, improved stability of drugs, improved bioavailability, and targeted delivery at the site of action. Thus, this review article aims to provide the mechanistic involvement of microglial activation in AD and current updates of various microglial-based therapies and nano carrier-based approaches to treat and manage AD.

## 2. Molecular Pathogenesis of AD

**Figure 1:**
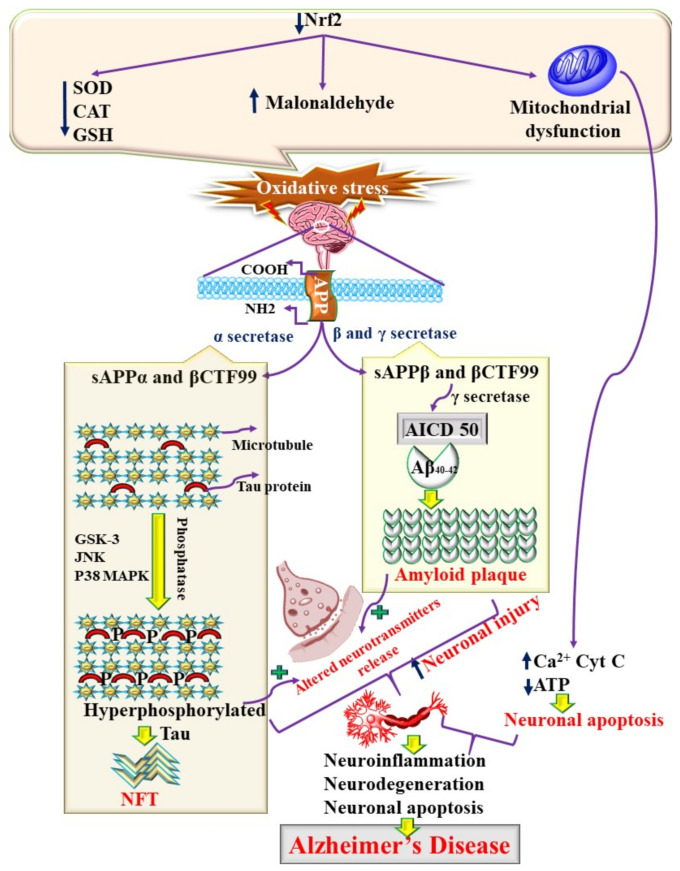
Molecular mechanisms of AD, involving oxidative stress and mitochondrial dysfunction.

AD is a complex disease and multifactorial in origin [10]. Various contributing attributes, such as increased oxidative stress, abnormal mitochondrial function, endoplasmic reticulum stress, neuroinflammation, the production of Aβ, and hyperphosphorylated tau, are involved in its pathogenesis [2]. Increased oxidative stress is considered the primary contributor to the pathogenesis of AD [11]. Oxidative stress is the result of excess reactive oxygen species (ROS) production. ROS are produced in normal physiological conditions, such as during cellular metabolism in the mitochondria, and in the diseased state excess ROS are produced [12]. Either dysfunctional mitochondria or the reduced activity of endogenous antioxidants, such as superoxide dismutase (SOD), glutathione (GSH), and catalase (CAT), is accountable for the production of ROS [12]. When an excess of ROS is produced, it causes oxidation of lipids, produces malonaldehyde (MDA), damages DNA, and modulates the production of peroxy-nitrite (ONOO) by interfering with the transcription of inducible nitric oxide synthetase (iNOS). Thus, increased ROS, iNOS, and ONOO, leading to the production of reactive nitrogen species (RNS), contribute to the pathogenesis of AD [11]. Increased ROS and RNS have been reported to initiate neuroinflammatory pathways (NF-kB/TLR-4/TNF-/p38 MAPK), modulate the NLRP-3 inflammasome, cause glial cell activation, and stimulate the production of Aβ and NFT via modulation of the GSK-3β/Wnt/JNK/Nrf2 signaling pathways [13,14,15]. In particular, the increased oxidative stress and nitrative stress interact with the amyloid precursor protein (APP) and enzymes involved in the production of Aβ and hence modulate the production and deposition of Aβ [16]. In the normally functioning brain, Aβ is produced, performs various neurophysiological functions, and is cleared from the brain via multiple mechanisms. APP is considered the key regulator in the maintenance of the balance between production and clearance. APP, along with α, β, and γ secretase, are involved in the homeostasis of Aβ [6]. β and γ secretase are responsible for the production of Aβ. Low-density lipoprotein receptor-related protein (LRP-1) is responsible for removing Aβ from the brain and transporting it into the systemic circulation, where it is excreted via the renal and hepatic metabolic pathways [17]. However, the increased ROS and RNS, on the one hand, cause the production of Aβ via stimulating the catalytic activity of β and γ secretase, whereas, on the other hand, the increase restricts LPR-mediated Aβ excretion and thus, increases the production and deposition of Aβ, as shown in Figure 1 [18].

## 3. Innate Immunity in AD

### 3.1. Glial Cells and Innate Immunity in AD

It is well established that AD’s pathogenesis is complex and goes beyond the amyloidogenic hypothesis and NFT [19]. Recent evidence has shown the profound role of glial cells in the pathogenesis of AD [20]. Glial cells were identified in the 19th century, and at that time, their functions were identified as nerve glue, called ‘Nervenkitt’ in German. Glial cells (astrocytes and microglia) constitute approximately 66% of the brain’s total mass [21]. Microglia are the immune component of the brain and perform phagocytic functions. It is interesting to highlight that the origin of microglia in the brain is almost similar to the macrophage’s peripheral origin, and hence their functions also overlap [21]. When the brain encounters neurotoxins or is exposed to any pathological microbes, the microglia induce the production of damage-associated molecular patterns (DAMPs) and pathogen-associated molecular patterns (PAMPs), leading to the activation of NLRP3 and pro-inflammatory cytokines, which contributes to the progression of AD.

Thus, it can be understood that dysfunctional microglia leads to the pathogenesis of AD via a paradigm shift in its basic functional attributes [22]. Furthermore, several pieces of evidence have shown the involvement of dysfunctional and hyperactive microglial cells among AD patients [23]. Briefly, it can be concluded that the phagocytic microglia, in the case of AD, lose their potential to clear neurotoxic components, such as Aβ, and mediate neuroinflammation and neurodegeneration [23]. Furthermore, it is also found that, during the initial pathogenesis of AD, activated microglia are responsible for the deposition of Aβ [24].

### 3.2. Microglia and Neuroinflammation in AD

The neuroinflammatory aspects involving microglial activation have been extensively explored in clinical and preclinical findings. The outcome of various studies reveals the dual role of microglial activation [25]. M1 polarization has been linked with neuroinflammation, whereas M2 polarization has been linked with neuroprotection [25]. Microglial activation and its proliferation, in response to neurotoxic stimuli, causes the increased production of pro-inflammatory cytokines, ROS, RNS, and other neuroinflammation mediators, leading to reduced excretion of Aβ, increased deposition of Aβ, and the production of NFT [26]. Apart from the role of M1 polarized microglia (PM) in the production of pro-inflammatory cytokines, these microglia also cause neuroinflammation by modulating NLRP3 inflammasome pathways. It has been discussed previously that the microglia sense the microenvironment [27]. Thus, these cells also detect the presence of DAMPs and PAMPs and cause inflammasome activation leading to AD’s pathogenesis [27]. The pathological role of the NLRP3 inflammasome has been well identified in the clinical and preclinical models of AD [28]. In response to microglial activation, NLRP3 activates and converts procaspase-1 into caspase-1 via autocatalysis and activated caspase-1, converting pro-IL-1 into UL-1b, which is ultimately responsible for neuroinflammation and neuronal death [29,30].

**Figure 2:**
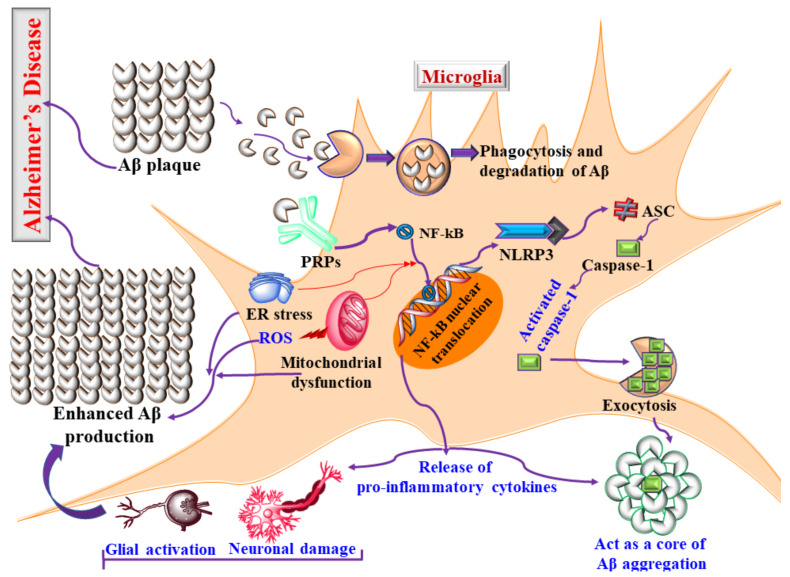
Mechanism of microglial activation, Aβ aggregation, neuroinflammation, and Alzheimer’s disease.

Additionally, deposited Aβ in the brain also causes the activation of the NLRP3 inflammasome and increases the level of IL-1β [31]. Interestingly, the activated inflammasome, apart from neuroinflammation, also induces the phosphorylation of tau protein and assists in producing NFT [31]. Thus, the NLRP-3 inflammasome is a link between Aβ and tau hyperphosphorylation, confirming the involvement of the NLRP-3 inflammasome in neuroinflammation and AD, as shown in Figure 2 [32].

### 3.3. Microglia and Tau Pathology

The previous section reported that activated microglia interact with Aβ and participate in AD pathology [33]. In this section, we will discuss the association of microglial activation and tau pathology in AD. The concept of microglial activation in tau pathology originated from a study by Virginia Lee’s group, where the administration of an immunosuppressant drug mitigated microglial activation to reduce tau phosphorylation [34]. Based on the outcome, it was proposed that microglial activation is accountable for tau pathology [34]. Later, it was found that microglial activation-mediated NLRP3 activation is the key molecular pathway in tau pathology [35]. Hence, inhibition of microglial activation can reduce the deposition of Aβ and tau pathology. However, along with the role of NLRP3, various other signaling molecules, such as p38 MAPK and GSK-3, and enzymes, such as phosphatase, under the influence of activated microglia, are also responsible for tau pathology, as shown in Figure 2 [36].

## 4. Therapeutic Approaches for the Management of Microglial-Activated AD

Recently, a better understanding of the role of microglial activation in AD has led to the exploration of three therapeutic alternatives: (1) the use of anti-inflammatory drugs to manage neuroinflammation; (2) modulating microglial polarization from M1 to M2 to achieve an anti-inflammatory effect; and (3) modulating the priming of microglia [15]. Among the various anti-inflammatory drugs, non-steroidal anti-inflammatory drugs (NSAIDs) are extensively used, but NSAIDs are ineffective based on the outcome of different clinical studies [37]. The primary reason for the ineffectiveness is proposed to be the non-specific suppression of inflammatory homeostasis. Minocycline is another drug explored for its possible anti-inflammatory effects. It was hypothesized that minocycline reduces NO, IL-1β, TNF-α, and mediators of inflammation and improves cognitive dysfunction by inhibiting microglial activation [38]. However, the outcome of the clinical trial showed no beneficial effect among AD patients [37].

Additionally, NSAIDs are associated with severe gastrointestinal side effects, ulceration, nausea, and hepatotoxicity, whereas the use of minocycline is associated with GI disturbance [39]. Thus, there is an unmet need for therapies targeting microglial activation, specifically the various downstream signaling pathways, such as NF-kB, NLRP3, caspase-1, and p38 MAPK, to reduce the production of microglial-mediated pro-inflammatory cytokines [40].

Some work has already been done to develop more targeted therapies. VX-765 (caspase-1 inhibitor), in the preclinical studies, showed a significant anti-Alzheimer’s effect via the inhibition of microglial activation, the reduction of Aβ deposition, and improving cognitive dysfunction [41]. Etanercept (TNF-α blocker) showed a moderate anti-Alzheimer’s effect in a small number of AD patients, which led to a phase II trial [42]. The outcome yielded a significant therapeutic effect. Hence, the phase III trial is proposed to begin shortly [43]. Similarly, the use of IL-1β receptor antibodies improves cognitive dysfunction and inhibits the formation of NFT in a preclinical study [44]. The use of IFNβ1a and resveratrol (sirtuin-1 agonist) also reduces the level of inflammatory markers in CSF, shows a significant anti-Alzheimer’s effect and improves cognitive function [45,46].

Apart from anti-inflammatory drugs, researchers have also explored the strategy of shifting M1 polarization towards M2. PPAR-γ is a well-known agent for shifting the polarized state from M1 to M2 or from pro-inflammatory microglia to anti-inflammatory microglia [47]. Rosiglitazone was explored for this purpose, and phase I and phase II trials showed significant clinical outcomes. Unfortunately, the outcome of the phase III trial was ineffective, and the possible reason was concluded to be the poor solubility and low bioavailability of the drug [48]. Similarly, an NLRP-3 inhibitor was also explored for promoting the polarized state of microglia. Preliminary studies show the reduced aggregation of Aβ, the reduced phosphorylation of tau protein, and improved cognitive function. However, the clinical efficacy of the NLRP3 inhibitor is yet to be validated in clinical studies [49].

Additionally, small molecules targeting microglial activation, such as COR388, Salsalate, AL002, and NP001, are under initial screening, whereas VX-745, Sargramostim, Valaciclovir, and Xanamem are part of a phase II clinical trial, and ALZT-OP1a is being screened in a phase III clinical trial. ALZT-OP1 is the combination of ibuprofen (NSAIDs) and cromolyn sodium (mast cell stabilizer). ALZT-OP1 is proposed to inhibit Aβ aggregation and stimulate the transition of M1 to M2, reducing neuroinflammation [5]. VX-745 is a small molecule that readily crosses the blood-brain barrier (BBB) and is a selective inhibitor of MAPKα. MAPKα is a well-established signaling molecule responsible for the production of pro-inflammatory cytokines and Aβ aggregation [50]. Additionally, MAPKα modulates receptors located on microglial cells and worsens the clinical attributes of AD [50]. GV-971 is an oligosaccharide derived from marine algae and has been approved in China to manage and treat AD-related cognitive dysfunction [51]. More details of the various drug candidates acting as either inhibitors of M1 microglial polarization or those that stimulate the shift of M1 to M2 phenotype are discussed below.

### 4.1. Drugs Targeting Inhibition of M1 Microglial Polarization

It is well established that M1/M2 balanced neuronal function is the prerequisite for the normal functioning of the central nervous system (CNS). Targeted therapies can either inhibit MI polarization or stimulate the transition from M1 to M2 [26]. The localization of receptors, such as cannabinoid receptor type 2(CB2) or Toll-like receptors (TLRs), at the surface of microglia, appears to be a novel mode of targeting microglial-induced neuroinflammation [52]. TLRs are an essential component of microglial-mediated neuroinflammation and AD [53]. Candesartan and cilxetil are two FDA-approved drugs for the management and treatment of hypertension and heart failure that show promising activity in their ability to inhibit TLR expression [54]. Moreover, an in vitro study showed that rifampicin had anti-inflammatory activity and reduced TLR2 mediated neuroinflammation [55]. Furthermore, TAK-242 and RSLA (TLR4 antagonist) exhibited significant anti-inflammatory activity, mitigated microglial activation, and reduced the production of TNF-α, and hence showed a neuroprotective effect [56]. β-caryophyllene and JWH133 are CB2 receptor agonists that inhibit microglial activation and exhibit anti-inflammatory and neuroprotective effects [57].

JAK/STAT and NF-kB are two critical pathways involved in the pathogenesis of AD and neuroinflammation. Studies have shown their correlation with microglial activation, and hence, these pathways offer a target for inhibiting microglial activation [58]. α-asarone and tanshinone-I are two naturally occurring bioactive compounds involved in the modulation of the NF-kB pathway and inactivation of microglia (inhibition of M1 polarization) [59]. These bioactive compounds reduce the production of pro-inflammatory cytokines, such as TNF-α, IL-6, and IL-1β, and increase the production of IL-10 and hence exhibit neuroprotective effects [60]. Apart from these natural bioactives, apocynin and resveratrol inhibit microglial activation and show significant antioxidant and anti-inflammatory activities via targeting NADPH oxidase [61]. Additionally, compounds, including lenalidomide, zonisamide, minocycline, curcumin, ginsenoside, piperine, rosmeric acid, curcumin, astilbin, etc., inactivate microglia, reduce the production of pro-inflammatory cytokines, and have an anti-Alzheimer’s effect [61]. Additional details of the various drugs responsible for M1 polarization are shown in Table 1.

### 4.2. Drugs Targeting the M1 to M2 Phenotype Shift

Apart from inhibiting microglial activation, the phenotypic shift from M1 to M2 appears to serve as a potent alternative for the treatment and management of AD. Studies have shown that IL-10, cAMP, Vit D, etc., stimulate the phenotypic switch from M1 to M2 and exhibit significant neuroprotection [68,69,70]. IL-10 is a well-known and established anti-inflammatory cytokine responsible for inhibiting NF-kB-mediated neuroinflammation and reducing the expression of iNOS, leading to neuroprotection [71]. cAMP is an intracellular signaling molecule and is accountable for microglial transition under the influence of cAMP kinase. However, this transition is only seen when cAMP is used in combination with IL-4 [72]. When this combination is used, M2 microglia mitigate ROS production and reduce pro-inflammatory cytokines [73]. Additionally, cAMP analogs, adenyl cyclase stimulators, or PDE inhibitors, such as sildenafil, yonkenafil, ibudilast, etc., induce the M1 to M2 transition and exhibit significant neuroprotection [74,75,76]. Vitamin D is known to exhibit substantial antioxidant and anti-inflammatory effects via an M1 to M2 shift as well as inhibition of M1 microglial polarization [59]. Studies have shown that vitamin D reduces the expression of iNOS, TLR-4, and increases IL-10, IL-4, CCL17, TGFβ, CD-163, CD-204, and CD-206 [70]. Based on preclinical studies, PPARγ is involved in the etiology of neurodegenerative disease, and reduced PPARγ expression is related to the pathogenesis of AD [77]. Pioglitazone and rosiglitazone (PPARγ agonists) inhibit microglial activation and stimulate the M1 to M2 phenotypic shift, leading to significant anti-inflammatory and neuroprotective effects [77,78]. Additional details regarding the various drugs responsible for the phenotypic transition from M1 to M2 are shown in Table 2.

### 4.3. Limitations of Existing Targeted Drugs to Combat AD

No doubt, as mentioned above, the novel and repurposed drugs are potential candidates to target microglial activation either via the inhibition of M1 microglia activation or via the transition of M1 to M2 macrophages. However, these drugs possess pharmacological as well as pharmacokinetic limitations [80]. The pharmacological limitations can be understood in terms of their systemic side effects on vital organs. The pharmacokinetic limitations can be understood in terms of their fast hepatic metabolism and permeation across the BBB [81,82].

### 4.4. Natural Bioactives as Potential Modulators of Microglial-Mediated AD

Along with several synthetic novel small molecules targeting microglia, various natural bioactives have also been explored for their possible anti-Alzheimer’s effects [7,83,84,85]. These natural bioactive compounds offer certain benefits over synthetic drugs, including ease of availability, low price, and multifactorial mechanisms of action. However, these bioactive molecules are limited due to the lack of a well-validated safety profile and regulatory approval [86]. Notably, the pharmacokinetic limitation of crossing the BBB is also a significant restriction of these bioactive compounds [86]. However, the pharmacokinetic limitation of synthetic drugs and natural bioactive can be overcome by using nanotechnology and incorporating these drug candidates in various nanocarriers [82]. Some of the natural bioactive compounds explored for targeting microglial-mediated AD are discussed in Table 3.

## 5. Nanocarrier as a Potential Tool for Groundbreaking Drug Delivery in AD

### 5.1. Targeted Drug Delivery into the CNS and Challenges to Cross the BBB

Currently, the available pharmacotherapeutics are used to mitigate microglial-related AD and cognitive dysfunction. The existing drugs face challenges in crossing the BBB in their stable form and cannot interact with proteins and receptors and inhibit microglial activation [107]. However, drug modifications, such as the pro-drug approach, can overcome these limitations and offer the advantage of bypassing systemic metabolism and ensuring active drugs at the site of action [108]. In addition to the pro-drug approach, engineered drug delivery is another novel method to overcome the limitations [30]. This approach offers the release of active drugs at the site of action via crossing the BBB, offers stable and sustained drug delivery, and enhances therapeutic outcomes [30]. Thus, it can be concluded that nanotechnology using nanocarriers offers multiple advantages in crossing the BBB and can exhibit desired pharmacological attributes [109]. According to ECR, nanocarriers are 1–100 nm in size [110]. Nanocarriers are advantageous, offering a greater surface area that eventually increases the drug loading capacity, increases the interaction of the drug with the receptors and other target proteins, and reduces the side effects [110,111].

Nanocarriers for targeting microglial activation can be prepared by altering their surface charge, particle size, particle size chemistry, and through the use of various ligands [112,113]. Nanocarriers are broadly classified into organic and inorganic, based on the material composition [113]. Additionally, various vehicles are also used for targeted drug delivery. For instance, iron-oxide-based nanocarriers are used to treat brain tumors via their magnetic property [114]. Recently, engineered exosomes in conjugation with nanocarriers have been explored for targeted drug delivery. The exosome offers a much lesser immune reaction, crosses the BBB, and becomes degraded, thus providing a biodegradable property [115].

Furthermore, gold nanoparticles (NPs) cross the BBB via passive diffusion, whereas silver and titanium dioxide NPs disrupt tight junctions [116,117]. Several other methods exist for disrupting the BBB permeability, such as ultrasonic waves and hyperosmotic agents [118,119]. However, using such methods to enable NPs to cross the BBB has certain limitations, such as allowing some neurotoxic agents into the CNS, which might alter the brain’s normal functioning [119].

Various positively charged NPs have been explored for their possible interaction with the negative surface of brain capillary endothelial cells (BCECs) and for crossing the BBB transcytosis [120]. For example, chitosan or polyethyleneimine have been used for targeted delivery into the brain [120,121]. These positive polymers are considered ideal for delivering nucleic acids or other negatively charged drugs, as NPs allow for easy assembly and reach the action site [122].

Moreover, studies show that insulin and some antibodies of insulin receptors can be utilized to target the BBB [123]. The transferrin receptor (TfR) is one of the extensively explored receptors in the BBB for targeting. Hence, NPs conjugated with transferrin have successfully been used for targeted delivery into the brain [124,125,126]. Studies also report that multiple fabrication mechanisms are advantageous over single fabricated NPs, and hence magnetic NPs in conjugation with transferrin were explored. The outcome showed significant increases in its concentration in the brain [127].

Similarly, NPs in conjugation with chitosan and bradykinin B2 antibodies were explored for targeted delivery into the brain [102]. Apart from targeted drug delivery for modulation of microglial activation, intranasal (IN) drug delivery also appears to be a promising approach. IN drug delivery offers the advantage of bypassing the systemic circulation and increasing drug bioavailability into the CNS via the nasal epithelium olfactory bulb. However, despite significant benefits, certain limitations exist, such as low drug loading capacity, nasal irritation, and drug degradation in the nasal cavity. Hence, to overcome such limitations, polymers such as polyethylene glycol or other polymers are used [109].

### 5.2. Microglia and the Uptake of Nanocarriers

To target microglial cells using nanocarriers, it is important to understand the mechanism of their uptake. It has been reported that nanocarriers interact with the cell membrane of microglia and get internalized via endocytosis [128]. Endocytosis is classified into phagocytosis and pinocytosis, where pinocytosis is further subclassified into clathrin-mediated, caveolin-mediated, clathrin-caveolin-independent endocytosis, and micropinocytosis [129]. Published evidence shows a superior intake of dendrimers (4–10 nm in size) when the microglial cells are activated by exposure to lipopolysaccharides [130,131]. The physiochemical attributes, such as size, surface charge, and shape, are the critical parameters that affect the binding to microglia and their uptake. Various modifications, such as ‘protein corona,’ ‘urchin-shaped coating,’ and ‘bumps and thorn,’ have shown increased uptake by the microglial cells and offer a novel mechanism of targeting microglial activation [132,133,134].

### 5.3. Mechanism of Nanocarriers for Targeting Microglial Activation in AD

As discussed above, advancements in nanotechnology have made it possible to fabricate nanocarriers that cross the BBB and inhibit microglial activation [135]. The surface of microglial cells has different types of receptors, and by tailoring the nanocarriers in conjugation, drug candidates can modulate these receptors and their downstream signaling pathways [136]. Pattern recognition receptors (PRRs) are among the extensively studied receptors present on the surface of microglia and are responsible for the association and aggregation of Aβ [137]. TLRs and advanced glycation endproducts (RAGE) are among the PRRs, and the use of drug-loaded liposomes has shown to inhibit microglial-mediated neuroinflammation via modulation of these receptors [138]. SR-A1 and CD36 are also PRR members and play a pivotal role in Aβ aggregation. Polystyrene-based and silver nanocarriers have been shown to interact with these receptors and inhibit microglial-mediated neuroinflammation [139]. Veglianese et al. explored the role of PEG-conjugated nanocarriers in the delivery of minocycline across the BBB, demonstrating that these nanocarriers significantly inhibited microglial activation and reduced microglial activated neuroinflammation [140]. Another study explored the role of polymethylmethacrylate conjugated nanocarriers, revealing a significant permeation across the BBB and inhibition of microglial activation [140]. Zheng et al. investigated the role of resveratrol and selenium nanocarriers against Aβ-mediated neuronal stress and neuroinflammation and showed that the nanocarriers significantly reversed the neurotoxic manifestations [141]. Ren et al. used quantum dot nanocarriers to target dysfunctional mitochondria. These inhibited microglial activation and reduced oxidative stress and Aβ aggregation [142].

Studies have also shown that nanocarriers, because of their hydrophilic-lipophilic balance (HLB), avoid opsonization, have an increased zeta potential, and the repulsive force between particles increases significantly. This results in a stable and uniform distribution of nanocarriers in the neuronal tissue and exhibits an enhanced pharmacological effect. Mechanistically, nanocarriers exhibit the anti-Alzheimer’s effect via modulating the various surface receptors located on the microglial membrane and thus showed significant anti-inflammatory activity. Nanocarriers have been reported to block PRRs, TLRs receptors, RAGE, SR-A1, and CD36. Moreover, nanocarriers target dysfunctional mitochondria, counteract oxidative stress, and, by ligand modification, inhibit Aβ aggregation. Thus, it can be concluded that nanocarriers, when conjugated with FDA-approved drugs, such as donepezil and galantamine rivastigmine, inhibit AchE activity, increase Ach levels, improvise cognitive dysfunction, and slow down the progression of AD. However, nanocarriers, in conjugation with small molecules, act as anti-inflammatory agents by inhibiting microglial activation. When these nanocarriers are conjugated with natural bioactive compounds, they exhibit anti-Alzheimer’s effects via multifactorial mechanisms, such as antioxidant, anti-inflammatory, and anti-apoptotic modes, and by reducing Aβ. Thus, recently a phase II clinical trial for APH 1105 nanocarrier, an α-secretase modulator, i.e., APP inhibitor) was initiated to explore the beneficial role of nanocarriers.

**Figure 3:**
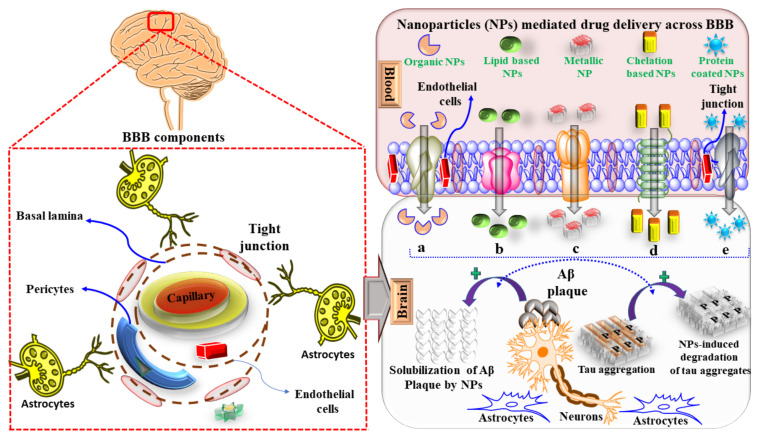
Mechanism of nanoparticles mediated drug delivery across BBB. (**a**) Transport of nanoparticles (NPs) via adsorption-mediated transcytosis pathway (**b**) transport of NPs via receptor-arbitrated transcytosis pathway (**c**) transport of NPs via cellular transport pathway (**d**) transport of NPs via transcellular pathway (**e**) transport of NPs via tight junctions.

Despite being a potential therapeutic modality, nanocarriers suffer from certain major limitations. When nanocarriers are fabricated by altering the surface morphology and conjugating them with various chemical moieties, unintended interactions with proteins, receptors, and cellular structures occur, resulting in neurotoxicity instead of neuroprotection [143]. However, it can be concluded that nanocarriers could be a novel approach to enhance the permeability of drugs across the BBB and selectively target microglia activation and treat AD, as shown in Figure 3.

## 6. Nanocarrier Based Drug Delivery for the Treatment of AD

### 6.1. Polymeric-Based NPs

Polymeric biodegradable NPs (PBNPs) are extensively used in the targeted drug delivery of small molecules in AD. One published study showed a significant improvement in cognitive dysfunction when PEGlyted PBNPs were used in AD [144]. In addition, PEGylated PBNPs reduced the Aβ peptide and ameliorated the pathogenicity of AD [145]. Memantine is one FDA-approved drug for the management and treatment of AD. PBNPs loaded with memantine showed significant anti-inflammatory and anti-Alzheimer’s effects [146]. Vitamin D has been extensively explored for its neuroprotective effect, but its low solubility and bioavailability are significant constraints [147]. Recently, poly lactic-co-glycolic acid (PLGA) loaded vitamin D was tested in a murine AD model and reduced neuronal apoptosis and neuroinflammation and improved cognitive function [147]. Zinc and sitagliptin-loaded NPs were explored for their possible anti-Alzheimer’s effect and were shown to improve cognitive dysfunction and alleviate neuroinflammation [148,149]. Acetylcholine esterase inhibitors are considered as first-line therapy for AD. Huperzine A was loaded into PLGA and was further conjugated with lactoferrin. The formulation exhibited improved release kinetics and considerably mitigated the symptoms of AD [150]. Similarly, Perlerin and Q10 loaded PBNPs showed a marked safety profile and potent anti-Alzheimer effects [151].

### 6.2. Nanomicellar

Nanomicellar (NM) are self-assembling nanosystems that are composed of a small head (hydrophobic) and a long tail (hydrophilic) [152]. NMs are designed so that the head remains in contact with hydrophobic drugs and the tail remains in contact with the aqueous phase [152]. Thus, NMs can be encapsulated in hydrophobic and hydrophilic drugs and further exhibit the advantages of being small in size, stable, possessing a higher drug loading efficacy, and can be conjugated with a wide range of ligands [153]. Q10 entrapped into NMs was administered orally by mixing it into the drinking water of AD challenged mice. Long-term administration of these NMs improved the behavioral and memory dysfunction and reduced Aβ plaques [154]. Incorporating curcumin into the NMs significantly improves its bioavailability and efficacy in mice [155]. Recently, ceramide nanomicelles and curcumin were studied. The use of these nanomicelles degraded the tau protein and induced autophagy that cumulatively resulted in an anti-Alzheimer’s effect [156].

### 6.3. Dendrimers and Nanogels

Dendrimers are monodispersed macromolecules with a branched 3D structural layout and offer an advantage for the conjugation of a wide range of ligands that can selectively target the site of action [157]. Lactoferrin conjugated with memantine dendrimers was explored for its therapeutic effect in AD challenged mice [158]. The study’s findings showed improved stability and cognitive dysfunction [158]. The administration of tacrine with polyamidoamine dendrimers showed a significant reduction in the toxicity of tacrine and exhibited a potential anti-Alzheimer’s effect [159]. Patil et al. explored sialic acid conjugated with polyamidoamine dendrimers and showed that its administration mitigated the Aβ-induced neuroinflammation, neuronal apoptosis, and other symptoms of AD [160].

In AD related to microglial activation, nanogels have shown promising effects in holding various drug candidates and macromolecules [161]. In one recently published report, deferoxamine nanogels and chitosan showed a significant anti-Alzheimer’s effect [162]. Ikeda et al. showed the anti-Alzheimer’s effect of pullulan and cholesterol nanogels via inhibition of Aβ and NFT [163]. Similarly, another novel study used a nose to brain drug delivery of insulin nanogels and showed a significant anti-Alzheimer’s effect [164].

### 6.4. Lipid-Based NPs for AD Therapy

Lipid-based nanocarriers are considered a superior carrier system for targeted drug delivery into the CNS. Lipid-based nanocarriers are more stable, offer excellent drug loading capacity, and have a stable carrier system [145]. Lipid-based nanocarriers are preferred for the nose to brain delivery of drugs in managing and treating AD associated with microglial activation [165].

#### 6.4.1. Solid Lipid Nanoparticles

Solid lipid nanoparticles (SLNs) are one of the extensively explored lipid-based nanocarrier systems for targeted drug delivery into the brain [166]. One of the novel approaches in the treatment and management of AD is to inhibit p-glycoprotein via the specific targeting of MC11 on the endothelial cells of the brain. For this purpose, transferrin structured SLNs were explored, and the study’s outcome showed a significant anti-Alzheimer’s effect [167]. Donepezil is an FDA-approved drug for the treatment of AD [1]. However, this drug exhibits limitations, including systemic toxicity and low BBB permeation [1]. Thus, the SLN of donepezil was formulated and administered via the intranasal route to overcome the limitation. In another study by the same researchers, donepezil-encapsulated SLNs showed significant anti-Alzheimer’s effects [168]. Similar to donepezil-encapsulated SLNs, curcumin-loaded SLNs were prepared and tested in the Aβ_1–42_-induced AD and associated behavioral and cognitive dysfunction. The study’s outcome showed that curcumin-loaded SLNs significantly reduced the behavioral-cognitive dysfunction and reversed the level of various neurotransmitters towards normal [169]. Curcumin-loaded SLNs were also explored for their possible antioxidant effects against AD-induced oxidative stress in a rodent model. In additioin, another study by the same research group reported the increased pharmacokinetic and pharmacodynamic profile of curcumin-loaded SLNs in a preclinical model of AD [170,171].

#### 6.4.2. Liposomes

Liposomes are amphiphilic and self-assembling nanocarriers extensively explored for targeted drug delivery into the CNS [172]. Liposome offers the advantage of providing a surface that can be effectively modulated with the help of protein and cell-penetrating peptides [172]. Thus, it offers passage across the BBB and targeted and site-specific drug delivery approaches [173]. In one published report, glutathione-PEG-loaded liposomes showed enhanced drug uptake across the BBB [174]. Curcumin-loaded nanocarriers have also been explored for their ability to permeate across the BBB and their anti-Alzheimer’s effect [175]. Osthole is a small molecule and synthetic derivative of coumarin. Osthole is known for its protective effect against hippocampal neurons [176]. Thus, liposome-encapsulated osthole was studied for its pharmacokinetic as well as pharmacodynamic effect. The study showed its potential anti-Alzheimer’s effect [177].

#### 6.4.3. Niosomes

Niosome is among the extensively explored lipid-based nanocarrier systems for the effective delivery of drugs across the BBB [178]. Artemisia-absinthium-loaded niosomes were explored for their effective delivery into the CNS in a preclinical model of AD [179]. The study’s outcome showed degradation of Aβ and mitigation of AD pathology. Hence, these findings open an avenue for the delivery of drugs in AD [179]. In another study, pentamidine-chitosan-glutamate-loaded niosomes were formulated and administered via the intranasal route. The study revealed significant permeation across the BBB and an anti-Alzheimer’s effect [180]. Among the various causes of AD, folate deficiency is considered one of the major contributing factors to AD pathology [181]. Thus, folic acid-loaded niosomes were formulated and explored for their possible pharmacokinetic and pharmacodynamic effects [182]. Rivastigmine is an acetylcholine esterase inhibitor, and it is among the four FDA-approved anti-Alzheimer’s drugs. In an investigational study, rivastigmine-loaded niosomes were studied for their permeation across the BBB. The study demonstrated significant bioavailability and an anti-Alzheimer’s effect [183].

#### 6.4.4. Nanoemulsion and Cubosomes

Nanoemulsion and cubosomes are additional lipid-based nanocarrier systems for targeted drug delivery in AD. Nanoemulsions are known for maximizing the efficacy and targeted drug delivery in AD [184]. Memantine is an FDA-approved drug and is clinically used orally. Naringenin is a natural bioactive molecule with potent antioxidant, anti-inflammatory, anti-apoptotic, and anti-Alzheimer’s effects. However, this biomolecule suffers is limited by low solubility and low bioavailability. Hence, a nanoemulsion of naringenin was engineered and tested in a preclinical model of AD [185]. The study’s outcome showed a promising safety profile and an anti-Alzheimer’s effect via the prevention of amyloidogenesis [186]. Memantine is indicated to slow down the progression of AD. An intranasal delivery of a memantine nanoemulsion was explored for possible anti-Alzheimer’s effects [187]. This formulation bypassed the BBB and enhanced the anti-Alzheimer’s effect compared to the conventional formulation [187]. Like memantine, cubosomal loaded donepezil mucoadhesive was also prepared and studied for targeted delivery in the management and treatment of AD [160].

### 6.5. Metallic NPs

Metallic nanoparticles are among the emerging and thrust areas for target drug delivery in the AD [188]. Although all metallic nanoparticles are not feasible for drug delivery, based on published reports, gold, silver, selenium, and cerium NPs are potential candidates for targeted delivery in managing and treating AD [189].

#### 6.5.1. Selenium and Cerium NPs

The role of oxidative stress in AD is extensively studied, and any drug candidate that reduces the level of ROS in the brain appears to be a potent anti-Alzheimer’s drug [18]. Selenium (Se) is a micronutrient that possesses significant antioxidant properties [190]. Recently, Se NPs and Se-selenite NPs were prepared and showed a substantial antioxidant effect. Hence, these can be further explored for potential anti-Alzheimer’s effects [191]. In one study, Se NP in conjugation with sialic acid effectively crossed the BBB and inhibited the aggregation of Aβ [192]. Thus, Se NPs are considered a promising tool for targeted drug delivery in managing and treating AD [192]. Similar to Se, cerium (Ce) NPs also possess significant antioxidant and neuroprotective potential. Additionally, Ce is easily uptaken by BBB cells and possesses no neurotoxic effect [193]. The promising neuroprotective effect of Ce and Ce-NPs in conjugation with triphenylphosphonium (TPP) was examined in a preclinical model of AD. The study showed that Ce NP prevented neuronal death and exhibited an anti-Alzheimer’s effect by altering mitochondrial dynamics [194].

#### 6.5.2. Gold NPs

Gold (Au) NPs have been extensively explored for their neuroprotective and permeation properties across the BBB for AD treatment [195]. Recently Au-NPs in conjugation with glutathione were studied for possible anti-Alzheimer’s effect and were shown to inhibit Aβ aggregation and had a potent anti-Alzheimer’s effect [176]. Similarly, an intrahippocampal and intraperitoneal administration of Au-NPs improved the learning and behavioral activities in a mouse model [196]. Au loaded with anthocyanin demonstrates significant anti-inflammatory and anti-Aβ aggregatory properties [197]. Furthermore, the administration of Au-NPs reduces the level of AchE and exhibited significant anti-inflammatory and anti-Alzheimer’s effects [198,199].

### 6.6. NP-Chelation-Based AD Therapy

As discussed previously, oxidative stress is one of the major confounding factors in neurodegeneration and AD [200]. Based on preclinical and clinical findings, altered levels of iron, aluminum, copper, zinc, etc., were accountable for the oxidative stress, neurotoxicity, DNA damage, and pathological manifestation of AD [201]. Thus, to take care of this situation, metallic-chelator-based NPs were fabricated and tested for possible antioxidant and anti-Alzheimer’s effects. When Fe and Cu-based NP were synthesized for possible ion chelation and an anti-Alzheimer’s effect, there was enhanced solubilization of Aβ and an excellent safety profile [202]. Moreover, desferrioxamine-based NPs were also explored in a preclinical model. The use of this iron chelator effectively reduced Aβ, prevent neuron degeneration, and showed promising anti-Alzheimer’s and neuroprotective effects [203].

Studies have shown that Cu ions are involved in the formation of Aβ via modulation of the Amyloid precursor protein. Hence, the use of NPs based Cu chelators is proposed to significantly reduce the severity of AD [204]. Clioquinol (CQ), a well-known Cu ion chelator, was loaded into NPs and showed a marked reduction in the accumulation of Aβ and neurodegeneration [205]. In addition, an NP-iron chelator was synthesized and coated with polysorbate 80 so that the nanoformulation could easily cross the BBB [206]. Similarly, in other studies, chelators, such as xanthone derivatives, deferasirox, deferoxamine, iodochlorhydroxyquin, and tacrine, reduced AchE inhibited ROS production and showed potent anti-Alzheimer’s effects [207,208].

### 6.7. Protein and Antibody-Coated NPs

Recently, NPs coated with the proteins and antibodies are gaining attention in managing and treating AD [209]. For instance, NPs coated with serum albumin and loaded with R-flurbiprofen (a small molecule and anti-Alzheimer’s drug) showed excellent permeation across the BBB and exhibited improved anti-Alzheimer’s effects by reducing Aβ peptide levels in the brain [210]. Tacrine (an FDA-approved anti-Alzheimer’s drug) is potent, but its use is restricted because of its low BBB permeability and high hepatotoxicity [211]. Thus, serum albumin-coated NPs were used to transport tacrine, and the outcome of the study showed enhanced permeability across the BBB, mitigation of hepatotoxicity, and an anti-Alzheimer’s effect [212]. These protein-coated NPs not only act as potent therapeutic agents but have also shown promising results in the early diagnosis and onset of AD [213]. Apart from protein-coated NPs, antibody-coated NPs are also emerging as novel therapeutic approaches. Currently, immune-therapeutics have been extensively explored for possible anti-Alzheimer’s effects, but these drugs have been reported to cause meningoencephalitis [214]. Thus, antibodies coated with NPs were developed and explored for possible anti-Alzheimer’s effects [215]. Similarly, other studies also investigated the anti-Alzheimer’s effects of immunotherapeutic drugs, such as 83-14 monoclonal Ab and scFv-antibody coated with NPs, and the outcomes show favorable results [216,217].

## 7. Conclusions

AD is an extensively studied neurodegenerative disorder, and microglial activation plays a pivotal role in its pathogenesis [109]. Until now, the exact mechanism of microglial activation and AD is not understood. These microglial cells act as housekeepers during normal physiological conditions and engulf cellular debris, clear Aβ, and prevent its accumulation [6]. However, microglia get activated in response to neurotoxic chemicals, stress, trauma, and other coexisting diseases. Once activated, neuroinflammatory signaling pathways are modulated, and AD occurs. Studies have also shown that activated microglial cells alter synaptic coordination and neurotransmitter release, resulting in dementia and cognitive dysfunction [6]. Furthermore, it is necessary to understand that microglial cells exist in two dynamic states, M1 and M2, where M1 is pro-inflammatory and M2 is anti-inflammatory cells [218]. Therefore, therapeutic modalities either involve inhibiting M1 microglial activation or M2 microglial stimulation [218]. Currently, various small synthetic molecules, such as MCC950, AAV2-hIL-10, zonisamide, and JNJ7777120, and natural products, such as resveratrol, tanshinone-I, apocynin, etc., have been explored for inhibiting microglial activation. However, these small molecules and natural products are limited by fast hepatic metabolism and poor BBB permeation [219]. Nanotechnology offers the advantage over the conventional therapeutic approaches by stabilizing drugs to cross the BBB and exhibit a superior pharmacotherapeutic effect [220]. However, this is not always the case. For example, nanocarriers sometimes exhibit unintended interactions with the proteins and tissues due to their size, surface morphology, and neurotoxicity [221]. Cationic and metallic NPs, such as gold, silver, titanium dioxide, and silica NPs, interact with the proteins, disrupt cellular structures, alter cell membrane permeability, and exhibit neurotoxicity [222]. To overcome this problem, incorporating antioxidants into the NPs is proposed as an alternative approach [223]. Unlike metallic NPs, polymeric NPs are more stable and offer controlled release and selective targeting profiles [224]. However, these nanocarriers aggregate and exhibit neurotoxicity. Thus, attempts have been made to control their size, charge, and morphology to control these unwanted aggregatory properties [225]. Additionally, using PEG for surface coating and designing a microglial, specific ligand are considered better approaches to reduce the neurotoxicity of nanocarriers [226]. By doing so, a much lower dose of nanocarrier based-drug delivery will be required. This approach will also offer selective targeting to microglial receptors by their preferred uptake and bypass the interaction with cellular components within the CNS [226,227]. However, we suggest that more extensive in vitro characterization is required for these nanocarriers before exploring them in vivo. Additionally, different neuronal cell lines should be used to estimate their potential neurotoxicity and study cell viability, neuronal apoptosis, neuronal stress, and genotoxicity so that nanocarriers can move from the bench to the bedside for the management and treatment of AD.

## Figures and Tables

**Table 1:** Drugs responsible for M1 polarization.

S. No	Drug	Class of Drug/Target	MOA	References
[1].	Candesartan	TLR-2 inhibitor	Inhibit TL-2 and 4 expressions, inhibit microglial activation.	[54]
[2].	Rifampicin	TLR-2 inhibitor TLR-2 inhibitor	Reduces oxidative stress, level of TNF-a, and inactivates microglia.	[55]
[3].	TAK-242			[56]
[4].	β-caryophyllene	CB2 agonist	Inhibit microglial activation and exhibit an anti-inflammatory effect.	[57]
[5].	JWH133		Inhibit BBB damage, reduce iNOS expression, and level of pro-inflammatory cytokines.	[62]
[6].	α-asarone	Modulator of JAK/STAT or NF-kB pathways	Inhibit NF-kB activation, reduce the level of the pro-inflammatory cytokine, and improve behavioral function.	[59]
[7].	Tanshinone I	NADPH oxidase modulator	Microglial inactivation reduces oxidative stress and neuroinflammation.	[63,64]
[8].	Apocynin			
[9].	Resveratrol			
[10].	Diphenyleneiodonium			
[11].	Ghrelin	GHS-R1a ligand	Inhibit microglial activation, reduces the level of NO and ROS, and exhibit an anti-inflammatory effect.	[65]
[12].	JNJ7777120	Antagonist of H4R	Exhibit anti-inflammatory effect and improve behavioral dysfunction.	[66]
[13].	MCC950	NLRP3 inhibitor	Inhibit microglial activation, prevent AB deposition, tau phosphorylation and improve behavioral dysfunction.	[67]

**Table 2:** Drugs responsible for the phenotypic transition of M1 to M2.

S. No	Drug	Class of Drug/Target	MOA	References
[1].	AAV2-hIL-10	IL-10 agonist	Inhibit iNOS and NF-kB expression, and exhibit an anti-inflammatory effect.	[79]
[2].	Rolipram	PDE-4 inhibitor	Increases the level of cyclic AMP, reduces oxidative stress, level of TNF-a, and improves the phagocytic activity of microglia.	[74]
[3].	Sildenafil			[75]
[4].	Yonkenafil			[76]
[5].	Vitamin D	ERK inhibitor	Stimulate polarization state from M1 to M2. As a result, it inhibits M1 activation, reduces neuroinflammation, and improves cognitive dysfunctions.	[70]
[6].	Pioglitazone	PPAR-g agonists	Shift M1 to M2 polarization inhibit NF-kB activation, reduces iNOS expression and inhibits TLR-4 activation.	[77]
[7].	Rosiglitazone			[78]

**Table 3:** Details of natural bioactive targeting microglial cells in AD.

S. No	Drug	Class/Source	MOA	References
[1].	Magnolol	Lignan/*Magnolia officialis*	Stimulate microglial mediated phagocytosis of degradation of Aβ.	[87]
[2].	Naringenin	Flavanone/Grapefruit	Stimulate the shift of M1 to M2. Inhibit M1 activation, Aβ aggregation, and improved cognitive dysfunction.	[88]
[3].	Sarsasapogenin AA13	Saponin *Rhizoma Anemarrhenae*	Mitigate the AD-induced neuroinflammation via promoting microglial phagocytosis and inhibit the Aβ aggregation.	[89]
[4].	Eriodictyol	Flavanone/*Yerba santa*	Inhibit NF-kB/p38 MAPK and activate SIRT1 pathway.	[90]
[5].	Apigenin	Flavone/Fruits	Reduced the level of PGE2 and NO. Inhibit p38 MAPK/JNK/ERK1/2 pathways.	[91]
[6].	Dihydromyricetin	flavonoids/*Hovenia dulcis*	Inhibit the microglial mediated neuroinflammation via inhibition of JAK/STAT/NLRP3 pathways.	[92]
[7].	Icariside II	Flavonoid/*Epimedium brevicornum*	Inhibit the microglial mediated neuroinflammation via inhibition of TLR4/MyD88/NF-κB pathways.	[93]
[8].	Hesperidin	Bioflavonoid/citrus fruits	Inhibit the microglial mediated neuroinflammation via inhibition of NLRP3 and stimulate Akt/Nrf2 pathway.	[94]
[9].	Silibinin	Flavonolignan/*Silybum marianum*	Inhibit the neuroinflammatory activities of JNK/p38 MAPK/NF-kB pathway.	[95]
[10].	Safflower Yellow extract	*Carthamus tinctorius*	Reduce the expression of iNOS and increase the expression of arginase-1. Stimulate polarization state from M1 to M2. Inhibit M1 activation.	[96]
[11].	Curcumin	Polyphenol/*Curcuma longa*	Inhibit the microglia-mediated NF-kB pathway.	[97]
[12].	Ferulic acid	Grains, fruits, and vegetables	Inhibit the microglial-induced neuroinflammation via modulation of NLRP3/NFkB/MAPk/TLR4 pathways.	[98]
[13].	6-Shogaol	*Zingiber officinale*	Inhibit the neuroinflammation via modulation of NGF level.	[99]
[14].	Epigallocatechin-3gallate	Catechin/*Camellia sinensis*	Reduce the level of TNF-α, IL-6, IL-1β, stimulate Nrf2/HO pathways, and inhibit microglial activation.	[100]
[15].	Andrographolide	Diterpenoid/*Andrographis paniculata*	Inhibitor of MAPK pathway and restrict the nuclear translocation of NF-kB.	[101]
[16].	Andalucin	Sesquiterpene lactone/*Artemisia lannta*	Reduce the level of TNF-α, IL-6, IL-1β, stimulate Nrf2/HO pathways and inhibit microglial activation.	[102]
[17].	Oleanolic acid	Pentacyclic triterpenoid	Reduce the level of TNF-α, IL-6, IL-1β and exhibit the antioxidant effect. Inhibit microglial activation.	[103]
[18].	Piperlongumine	Alkaloid/*Piper longum*	Inhibitor of NF-kB pathway, restrict the activities of β and γ secretases and inhibit the aggregation of Aβ.	[104]
[19].	Geniposidic acid	Iridoid glucoside/*Eucommia ulmoides*	Inhibitor of NF-kB pathway reduces the expression of iNOS, reduces the aggregation of Aβ, and inhibits microglial activation.	[105]
[20].	Aromatic turmerone	Essential oil/*Curcuma longa*	Inhibitor of NF-kB, TLR-4, and stimulator of Nrf2 pathways.	[106]

## Data Availability

Not applicable.
